# A Young Man With Gravitational Urticarial Vasculitis

**DOI:** 10.5826/dpc.1003a56

**Published:** 2020-06-29

**Authors:** Ali Sadeghinia, Jafar Taghizade, Nooshin Dolatyabi, Amir-hooshang Ehsani, Pedram Noormohmadpour, Anahita Rostami

**Affiliations:** 1 Department of Dermatology, Razi Hospital, Tehran University of Medical Sciences, Tehran, Iran; 2 Department of Dermatopathology, Razi Hospital, Tehran University of Medical Sciences, Tehran, Iran

**Keywords:** gravitational urticarial vasculitis, physical urticaria

## Introduction

Urticarial vasculitis presents with urticarial wheals and/or angioedema, often with purpura and ecchymotic stain. It can be associated with physical urticaria and appears more frequently in the pressure sites [1]. Urticarial vasculitis is most often idiopathic, although it has been described in association with connective tissue diseases (systemic lupus erythematosus and Sjögren syndrome), neoplasia (monoclonal gammopathy and lymphoma), chronic viral infection, drugs, and serum sickness-like diseases [1].

Herein we report a case of gravitational urticarial vasculitis induced by standing and sitting more than 15 minutes.

## Case Presentation

**Figure 1:**
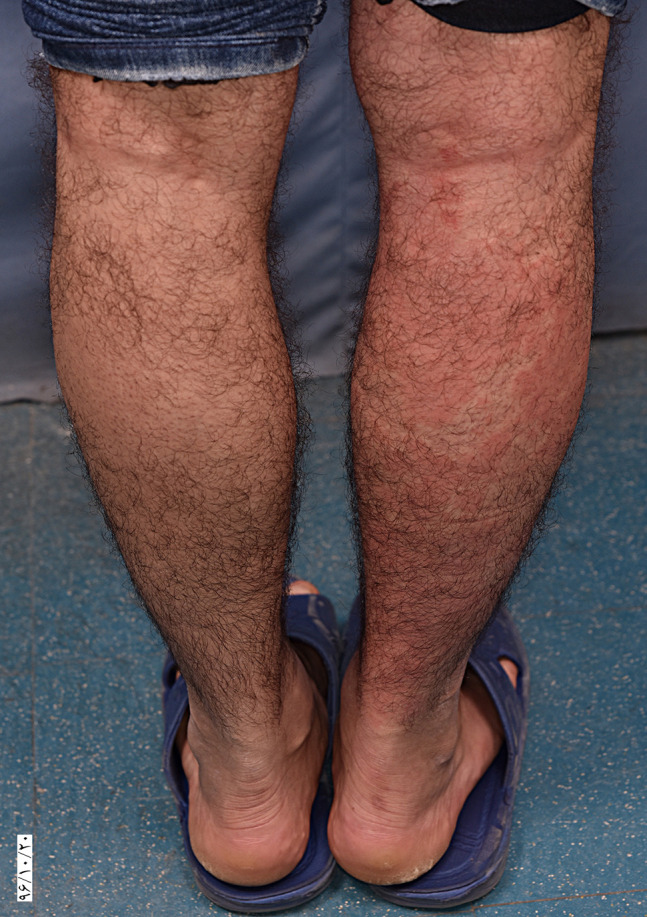
Development of urticarial plaques on the right leg after 15 minutes of the leg hanging.

**Figure 2:**
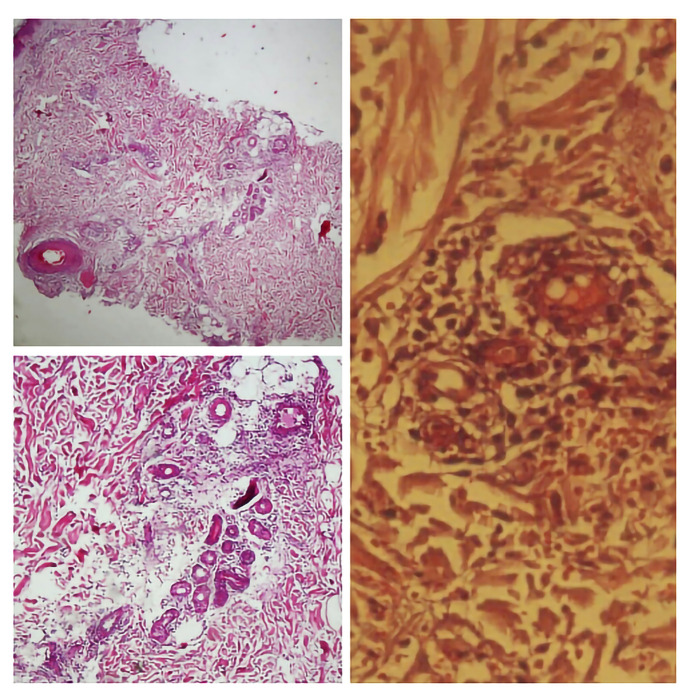
(A) Soft tissue edema within the deep dermis and around eccrine coil (H&E, ×4). (B) Lymphocytic and neutrophilic perivascular infiltration (H&E, ×10). (C) Fibrin deposition and extravasation of red blood cells in favor of small vessel vasculitis (H&E, ×40).

A 31-year-old man presented with painful and/or pruritic erythematous wheals on his legs after about 30 minutes standing, since childhood (6–7 years old). The wheals resolved within 3 to 4 days of rest. He did not complain of fever, arthritis, or malaise. His medical history was not significant. His father, aunt, and 2 cousins had similar symptoms and history, which improved after several years. As a provocation test, the patient was asked to hang one of his legs over the site of a chair and to bend the other leg. After 15 minutes, painful erythematous edematous plaques appeared on the hanging leg (Figure 1). A skin lesion punch biopsy revealed perivascular lymphocytic and neutrophilic infiltration extending to deep dermis and subcutaneous tissue and vasculitis involving small to medium-sized blood vessels (Figure 2).

Further evaluations including antinuclear antibody, antineutrophil cytoplasmic antibody, antiphospholipid, viral markers, serum complement, and cryoglobulin level were normal. The patient had received hydroxyzine, fexofenadine, and cetirizine, but they were ineffective, so we prescribed colchicine 1 mg/day, which was effective; the patient was lesion-free when he was on treatment. After 1 month’s treatment the patient discontinued colchicine and did not return for follow-up.

## Conclusions

It is known that urticarial vasculitis more frequently develops at pressure sites, but it is unusual to develop after standing or changing position [1]. Hirohata et al reported a case of postural cholinergic urticaria induced by a standing position, the same as our patient. Histopathological evaluation revealed mild perivascular lymphocytic infiltration, but there was no evidence of vasculitis [2].

This is a case of urticarial vasculitis induced by gravity with an unusual clinical presentation, which was managed by antineutrophil agents. We speculate that increasing intravascular pressure leads to microvascular endothelial damage and triggers extravasation of immune cells (including neutrophils and lymphocytes) and vascular damage in susceptible cases. Examination of further cases and recognition of clinical aspects will afford a better understanding of the underlying mechanisms.

## References

[1] Monroe EW (1981). Urticarial vasculitis: an updated review. J Am Acad Dermatol.

[2] Hirohata A, Yamaoka T, Hayashi M, Tani M, Katayama I (2016). Unique case of postural cholinergic urticaria induced by a standing position. Clin Exp Dermatol.

